# Chair squat performance as a potential predictor of nurses’ physical capabilities in ergonomic patient transfers

**DOI:** 10.1038/s41598-023-29968-0

**Published:** 2023-02-17

**Authors:** Anna Brinkmann, Christian Kowalski, Sandra Lau, Ole Meyer, Rebecca Diekmann, Andreas Hein

**Affiliations:** 1 Assistance Systems and Medical Device Technology, Carl von Ossietzky University of Oldenburg, 26129 Oldenburg, Germany; 2 Geriatric Medicine, Carl von Ossietzky University of Oldenburg, 26129 Oldenburg, Germany

**Keywords:** Musculoskeletal system, Occupational health, Risk factors

## Abstract

Muscle activation and movements performed during occupational work can lead to musculoskeletal disorders, one of the nursing profession's most significant health hazards. However, physical activity like exercise training tailored to the exposure and physical ability offers health prevention and rehabilitation. Professional nursing associations have advised squat training to promote occupational health because it strengthens lower limb and back muscles. Given that squatting is a fundamental part of many daily activities and various actions in caregiving processes, we hypothesized that chair squat performance is a potential predictor of nurses’ physical capabilities to perform occupational tasks. We conducted kinetic and electromyographic assessments of 289 chair squat repetitions and compared them to ergonomic patient transfer tasks. In this task, nurses transferred a supine patient to a lateral position in a care bed using similar movement characteristics of the squat task. This cross-sectional pilot study provides initial insights into nurses’ kinetic and muscle activation patterns of health-enhancing and compensational strategies. Highly asymmetric movements corresponded to distinct extremes in lower limb and spine muscle activity data—e.g., increased activity of the rectus femoris indicates increased hip flexion, including postural sway and, therefore, high torsional forces affecting the sacroiliac joints. The potential of the chair squat performance as a predictor of nurses’ physical capabilities in ergonomic patient transfers was quantified by a 2 × 2 contingency table resulting in an accuracy rate of 73%.

## Introduction

Physical activity promotes health, but muscle activation and movements performed during occupational duties can also lead to musculoskeletal disorders (MSD)^1^. MSD are among the most significant health hazards for professional employees and are a critical component of the global deterioration in mental health and physical performance^2–6^. Occupations with high physical work demands, such as patient handling in healthcare, display a high rate of long-term absenteeism and work disability due to musculoskeletal pain and MSD^2,4,7^. In this case, lower back pain is one of the main contributors to missed work and the most common occupational health problem^2–4,8–11^.

MSD and lower back pain risk increases with heavy work duties such as lifting and transferring patients frequently or in awkward, stooped, and forced postures^12,13^. Additional factors include asymmetrical lifting, holding, and moving patients, resulting in heavy backloading by compression, flexion, and rotation of the lumbar spine^5,6,8,10,14–16^. While musculoskeletal loads can be significantly reduced by using ergonomic working techniques as well as assistive devices and systems^8,9,12–17^, MSD are multifactorial, including both unmodifiable and modifiable factors. Genetic and morphological factors are unmodifiable, whereas psychosocial and biomechanical factors are modifiable—and thus targetable—to minimize the prevalence of MSD in nursing staff^13^. Previous research has implied that psychosocial and biomechanical risk factors can be reduced by individual intervention programs, including stress prevention training, to alter the individual amount and type of physical exposure, e.g., by redesigning job settings to increase the variety of nursing duties^13^. In addition, physical activity guidelines, such as those established by the WHO, recommend physical activity for musculoskeletal health, specifically for the working-age population^1,18,19^. Since there is a significant cross-sectional relationship between sickness absenteeism and lower muscle strength as well as obesity (BMI > 30 kg/m^2^), various studies have recommended physical activity and exercise tailored to alleviate MSD^1,18–20^. In addition, professional associations advise squat training to strengthen lower limb and back muscles^14,15,21–23^. In sum, a combination of factors is essential when targeting the modifiable factors of MSD to prevent and rehabilitate lower back pain among nursing staff. The first is to offer worksite-based and individually tailored physical exercise as well as training sessions on ergonomic working techniques. The second is to train nurses to use technical devices in caregiving processes^5,14,15,24^. However, these activities remain limited due to the lack of dissemination of interdisciplinary research knowledge^1^.

Our vision is to individually adapt worksite-tailored training exercises to the high physical stresses and strains endemic to care work. We hypothesize that the chair squat (CS) is a potential predictor of nurses’ physical ability in occupational duties such as ergonomic patient transfers (EPT). In this context, we assume that the analysis of lower limb and back muscle activity will highlight patterns of health-enhancing and compensational strategies. To assess this, we conducted kinetic and electromyographic (EMG) assessments in a laboratory setting to provide initial insights into the biomechanics and muscle activity in CS and EPT tasks of nurses. Specifically, to test our hypothesis, nurses were instructed to transfer a supine patient to a lateral position in a care bed using similar movement characteristics of the squat performance.

This cross-sectional pilot study serves as data collection to calculate the number of cases for future studies using effect size calculation to investigate whether the CS can be used as (1) a regular training exercise to (2) identify existing deficits in CS performance (since this would also take place during EPT) and to thus (3) conceptualize individualized training exercises based on CS. In the future, existing difficulties in CS performance could therefore be specifically trained to prevent potential health risks arising from EPT.

With this study, we contribute to the ongoing research on the physical relief of nurses through functional testing, providing interdisciplinary research knowledge for the design of exercises tailored to the individual and their worksite.

## Methods

### Participants

Healthy individuals with a nursing background participated in our study. Probands were recruited via mailing lists sent to addresses throughout the Carl von Ossietzky University of Oldenburg and various hospitals. Before participating in the study, each proband received verbal and written information about the content and purpose of the experiment. Each participant signed an informed consent form and provided written permission to publish identifiable images. A short questionnaire was used to collect demographic data and physical characteristics.

### Experimental design

The study was conducted in the AAL/Care Laboratory of the Carl von Ossietzky University of Oldenburg^25^ during the COVID-19 pandemic. All participants completed successive repetitive CS and EPT tasks. Participants were instructed to perform both tests on one force plate to measure external ground reaction forces (GRF) and moments while displacing the center of mass downward. In addition, non-invasive EMG measurements were used to record muscle activities of the lower limb and spine to gain information on the participants’ activation behavior. A multi-depth camera system^26^ was used to record the tasks performed three-dimensionally and connect them to the GRF and EMG data. The Commission for Research Impact Assessment and Ethics of the Carl von Ossietzky University of Oldenburg approved the study design (ethical vote: Drs.EK/2019/078). The study was conducted in accordance with the approved guidelines.

#### Chair squat

**Figure 1:**
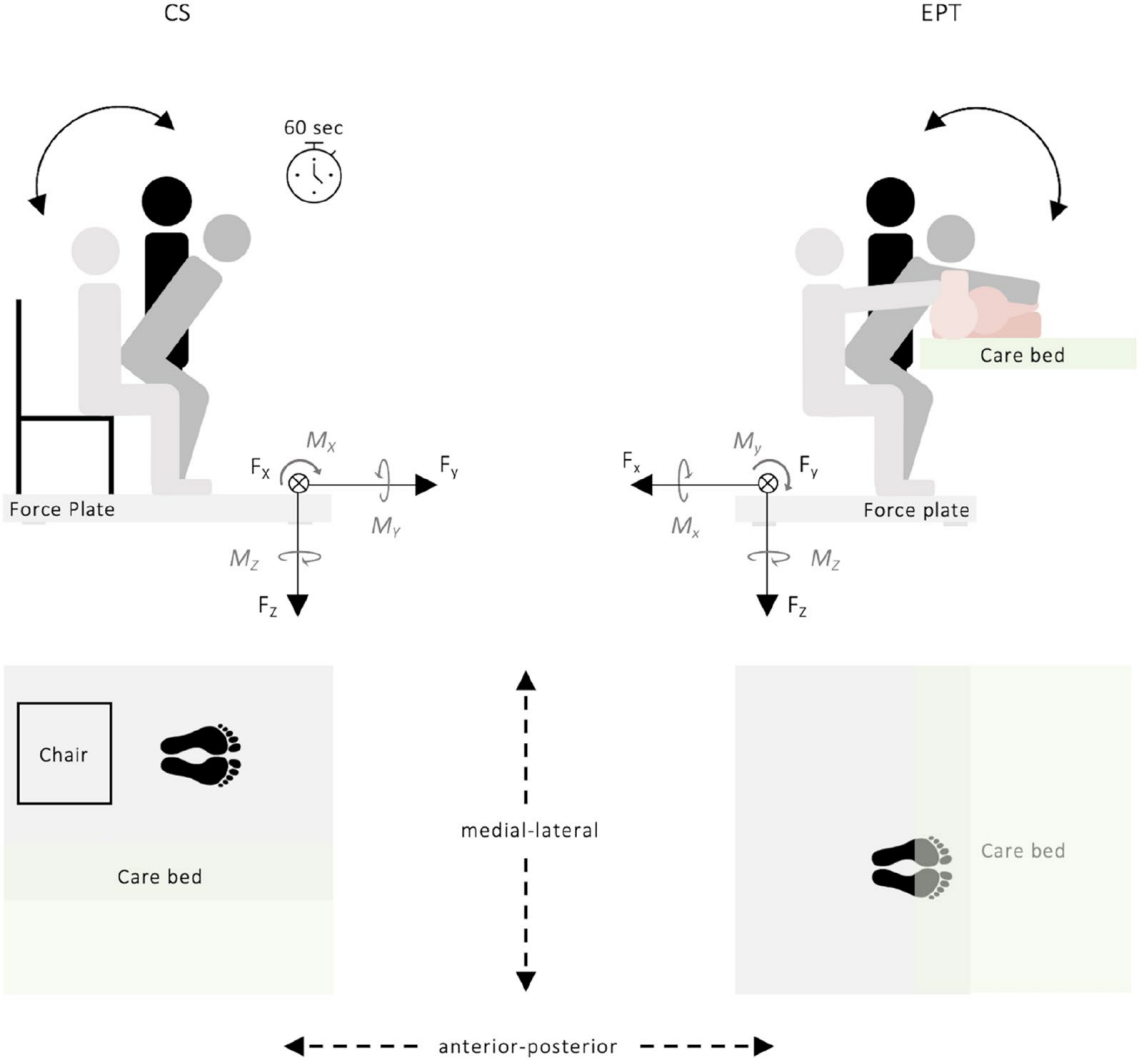
Schematic representation of the study configuration of the chair squat (CS) and the ergonomic patient transfer (EPT) task. F_X_, F_Y_, F_Z_ indicate ground reaction forces and their direction; M_X_, M_Y_, M_Z_ indicate moments and their direction.

The CS is an activity of daily living and covers several components of physical function, including balance, coordination, and lower limb strength^27,28^. The test was used as a bodyweight resistance exercise to reduce the risk of injury, standardize squat depth, and employ similar muscle groups as those used in EPT. Participants sat in the center of a standardized armless chair with a seat height of 45 cm (Fig. 1, CS). The initial position was a vertical trunk with a stretched back and a straight neck. The upper limbs were crossed with elbows bent toward the anterior part of the body. The feet were placed next to each other at shoulder width. The knee flexion angle was 90°. After a start command, each participant rose to full standing and squatted down with the buttocks touching the chair. Participants were instructed to perform the test without stepping off the force plate and to perform as many CS as possible in 60 s. The time was measured using a stopwatch, and the total number of CS was recorded in addition to kinetic and muscle activity data.

#### Ergonomic patient transfer

Changing the position of a patient from supine to lateral in a care bed is a frequent and firmly established activity in the daily occupational duties of nurses. This task is a prerequisite for transferring a patient from lying to sitting at the edge of the bed. For the experiment, a patient simulator (80 kg, 180 cm)^29^ was used for the transfer. All participants were instructed to perform the task from the left side of the care bed (Fig. 1, EPT) and not to step off the force plate or support themselves on the bed frame while conducting the task. Initially, each participant stood upright with both feet at shoulder width on the force plate facing the bed and the patient simulator (Fig. 1, EPT). The participants then inclined their trunks to the patient simulator and flexed their knees to shift to a stable position. The participants’ left hand touched the patient simulator’s pulled-up right knee, and the right hand was placed under the patient simulator’s right shoulder. Participants were instructed to ergonomically reposition the patient simulator using their leg muscles, instead of their spine muscles. Hence, they should flex their knees (90°) with a stretched neck and back to transfer the patient simulator ergonomically to its left side while displacing the center of mass downward (Fig. 1, EPT).

In addition, the task was performed conventionally to assess participants’ ability in EPT by comparison of force exertion^15,30^. For this purpose, each participant stood upright with both feet at shoulder width on the force plate facing the bed and the patient simulator, bending their trunks forward to grasp the patient simulator with both hands under its right shoulder to drag it sideways powerfully.

### Data recordings and analysis

#### Force plate

Force plate data were recorded using an AMTI Accupower system (AMTI, Watertown, MA, USA), consisting of a force plate and a specialized hardware and software system. Data acquisition took place at 200 Hz, displaying the GRF in medial–lateral (F_X_), anterior–posterior (F_Y_), and longitudinal (F_Z_) directions and the respective moments (M_X,_ M_Y,_ M_Z_). In addition, medial–lateral and anterior–posterior coordinates of the center of pressure (COP) were determined and used to measure balance control.

#### Surface electromyography

Surface EMG data were acquired from left and right erector spinae (ESL/ESR), gluteus maximus (GM), biceps femoris (BF), rectus femoris (RF), and vastus medialis (VM) of the lumbar spine and left lower limb. Before electrode placement, participants’ skin was shaved, abraded with fine sandpaper, and cleaned with alcohol. EMG signals were recorded using a device from Biovision (Biovision input box and DASYLab 4.010 software) and bipolar surface electrodes with a 10 mm inter-electrode distance (GE Medical/Hellige, 14 mm diameter). Local amplifiers were used for signal amplification (× 2500), and an analog-to-digital conversion unit sent the signals to the input box (National Instruments USB-6009, 14-bit) for further processing.

#### Data analysis

Data analysis was performed using custom-written functions in MATLAB (R2021a, MathWorks, Inc., Natick, MA, USA). The first step was visual inspection and elaboration using the recordings of the multi-depth camera system^26^ to precisely visualize participants’ postures and movement strategies and connect them to the force plate and EMG data. In the next step, both datasets were synchronized. Then, F_Z_ force plate data were normalized to each participant’s body mass and used to segment the EMG signals into EPT and CS squatting cycles. F_X_, F_Y_, and F_Z_ were combined into a resultant vector ($\left|\overrightarrow{r}\right|$), thus determining the maximum peak of every task conducted. Next, the resulting moments were analyzed in medial–lateral, anterior–posterior, and vertical directions due to flexing or twisting the trunk to the side. In addition, medial–lateral and anterior–posterior coordinates of the COP were calculated to quantify balance control by postural sway. Range values represent COP displacement during the CS and EPT. EMG data were rectified and smoothed by root mean square (RMS). Occasional baseline offsets were corrected after visual inspection of EMG data using an offset correction function. The datasets were divided accordingly into 60-s CS and EPT segments. A mean RMS signal was provided for each task and participant. Time axes were normalized to the period of the tasks using linear interpolation.

To differentiate participants’ ability in EPT, kinetic force plate data were used for group classification. Based on literature findings^15^ and previously conducted work^30^, we found that force exertion ($\left|\overrightarrow{r}\right|$) in manual patient handling is significantly reduced by considering ergonomic or biomechanical principles^30^. To this end, group classification was based on comparing the maximum peak of $\left|\overrightarrow{r}\right|$ when moving the patient simulator conventionally with the maximum peak of $\left|\overrightarrow{r}\right|$ in EPT for each participant. Those who reduced $\left|\overrightarrow{r}\right|$ in EPT were classified as one group (**A**), and all other participants were assigned to another group (**B**)^30^. The underlying methodology is described in Brinkmann et al.^30^.

### Statistical analysis

We used the Shapiro–Wilk test to verify the normal distribution of the data. Since the dataset included non-normally distributed data, we used the two-sided Wilcoxon signed-rank test and the Mann–Whitney test for non-parametric testing. Thus, significant changes in kinetic and muscle activity data were analyzed in each task. We performed inter-group and intra-group comparisons. Effect sizes (*r*) have been provided for both statistical tests. A *p*-value < 0.05 was considered statistically significant. In addition, we used a 2 × 2 contingency table as a statistical verification method to display the frequency distribution of the variables. It is used to evaluate chair squat performance as a potential predictor of nurses’ physical capabilities in EPT. Statistical analysis was performed using IBM’s SPSS Statistics software, version 27.0 (IBM, Armonk, NY, USA).

## Results

### Participants

Twelve healthy individuals with a nursing background participated in our study. One study participant was excluded from data analysis due to missing force plate data. Individual characteristics of the study population are summarized in Table 1. We analyzed a total of 289 CS (26.27 ± 4.22 in 60 s) from 11 study participants (two males) and compared them with their EPT performance.

**Table 1:**
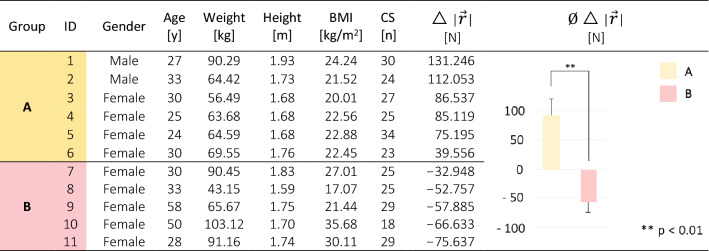
Demographic data, physical characteristics, and group classification of the study participants.

BMI = body mass index. CS = chair squat.

The deviation (△) between the maximum peak of the resultant ground reaction force vector ($\left|\overrightarrow{r}\right|$) of the conventional and ergonomic transfer is shown for each study participant and group^30^.

In the first step, we used kinetic force plate data to classify groups for further analysis of the CS and EPT^30^. Compared to the conventional transfer, six participants (two male) reduced $\left|\overrightarrow{r}\right|$ in EPT and were thus classified as group **A** (Table 1). The other five participants were classified as group **B**^30^. Group classification reached highly significant values for inter-group comparison in terms of minimizing $\left|\overrightarrow{r}\right|$ in EPT (*p* = 0.004, r = 0.826) (Table 1)^30^.

### Chair squat vs. ergonomic patient transfer

#### Force plate: moments

**Figure 2:**
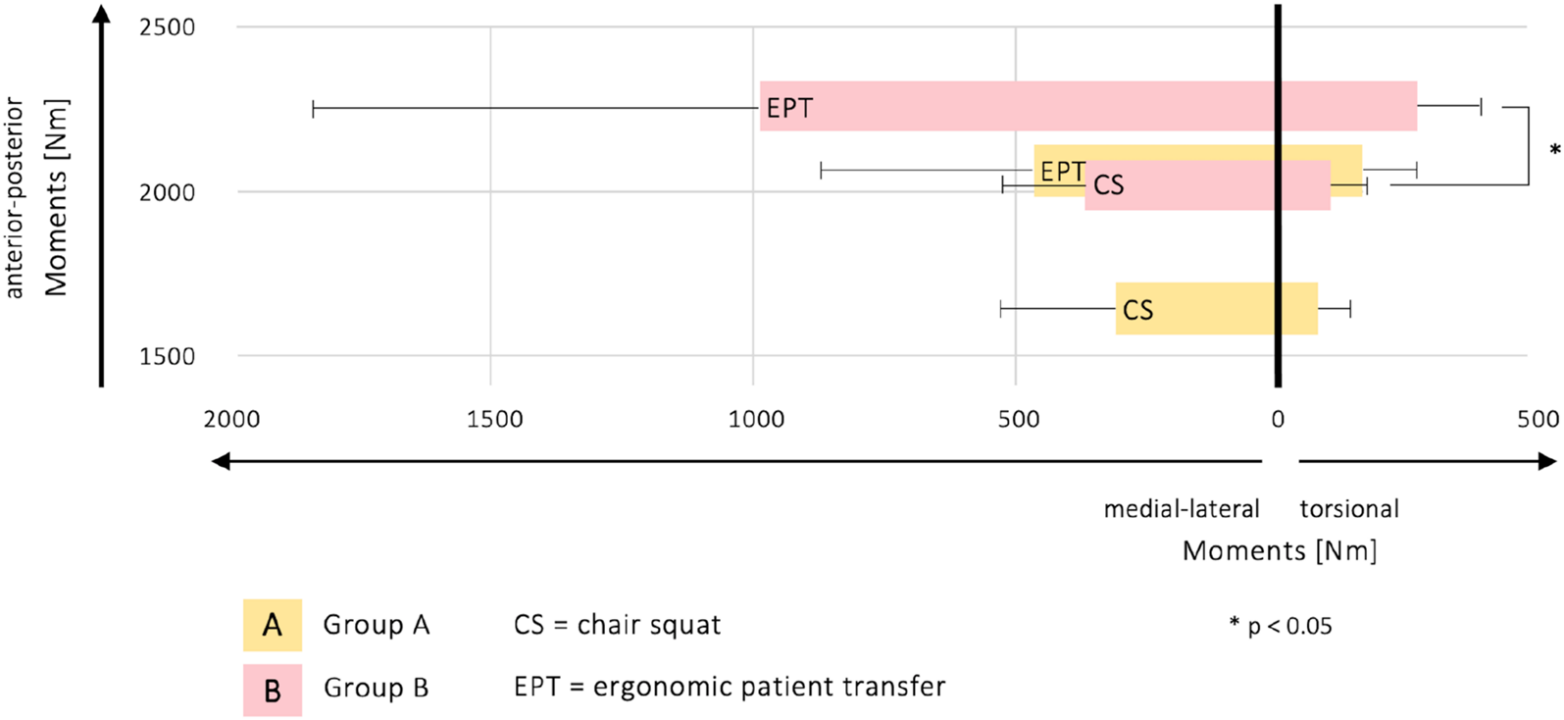
Representation of the medial–lateral and torsional moments for both groups and tasks as a function of trunk flexion (anterior–posterior moments). The results are presented as means ± standard deviations, including significant comparisons of each group’s two-sided Wilcoxon signed-rank test (Supplementary Material, Table 2). The inter-group comparison showed no statistical significance (Supplementary Material, Table 1).

We analyzed medial–lateral and torsional moments as a function of trunk flexion in the anterior–posterior direction to illustrate the level of asymmetry in the CS and EPT (Fig. 2). The left abscissa represents the moments in the medial–lateral direction, and the right abscissa shows torsional moments. The ordinate depicts moments in the anterior–posterior direction. The inter-group comparison was not statistically significant (Supplementary Material, Table 1). The main difference was found in the intra-group comparison of torsional moments among group **B** (Supplementary Material, Table 2).

#### Force plate: center of pressure

**Figure 3:**
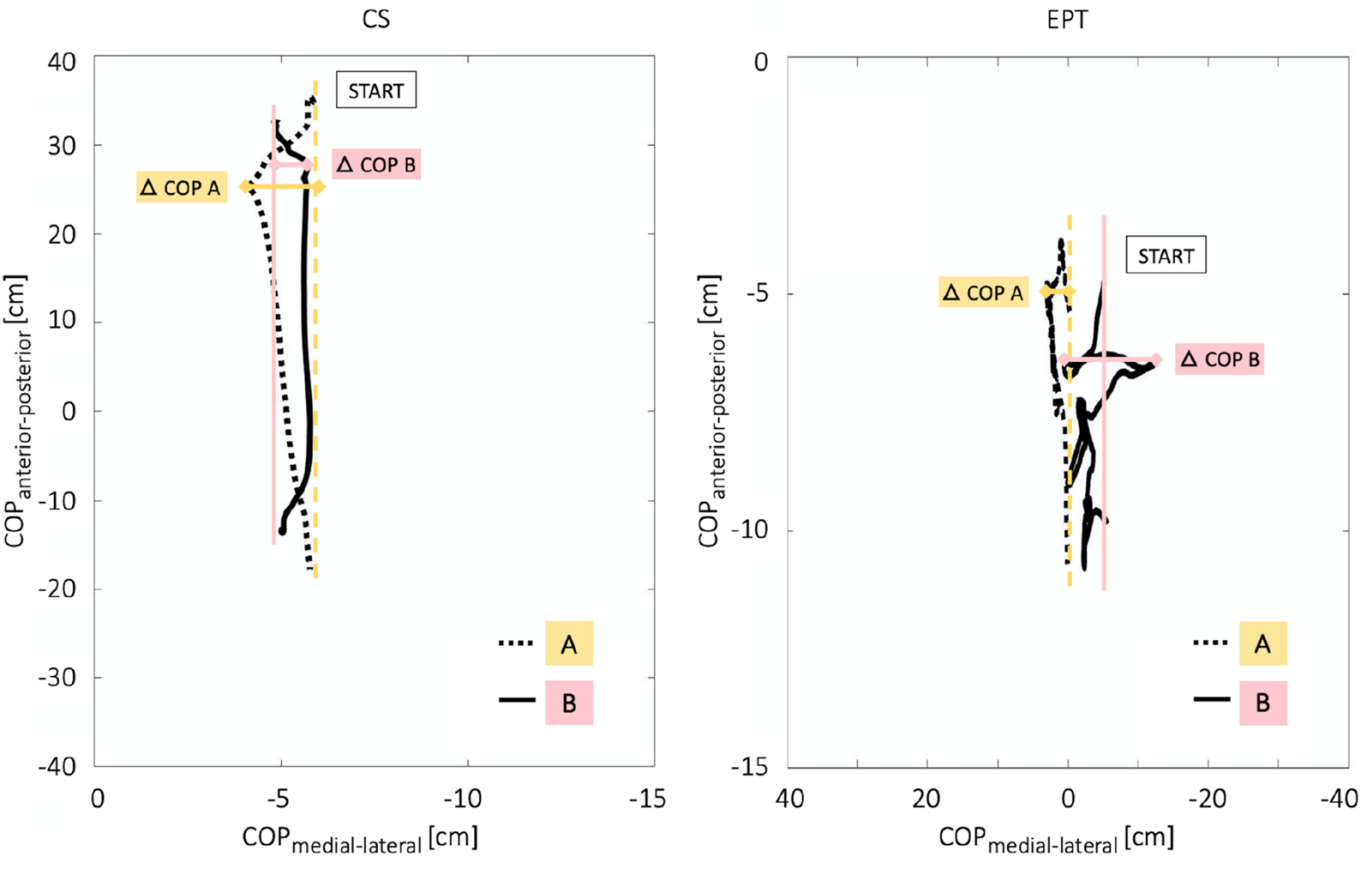
Representation of the postural sway determined by the COP in medial–lateral and anterior–posterior directions during the CS and EPT. The initial COP positions are indicated (START). Data are presented as means for both groups.

In addition, the postural sway determined by the COP in medial–lateral and anterior–posterior directions represents differences in balance control behavior. Respective range values indicate the mean sway in the CS and EPT. At the beginning of both tasks, the highest range values of both groups were found in the medial–lateral direction (Fig. 3). During CS, group **A** had a 1.660 ± 1.420 cm COP range, and group **B** had a 0.807 ± 6.180 cm COP range. During EPT, group **A** had a 2.886 ± 9.499 cm COP range, and group **B** had a 12.799 ± 18.851 cm COP range.

**Figure 4:**
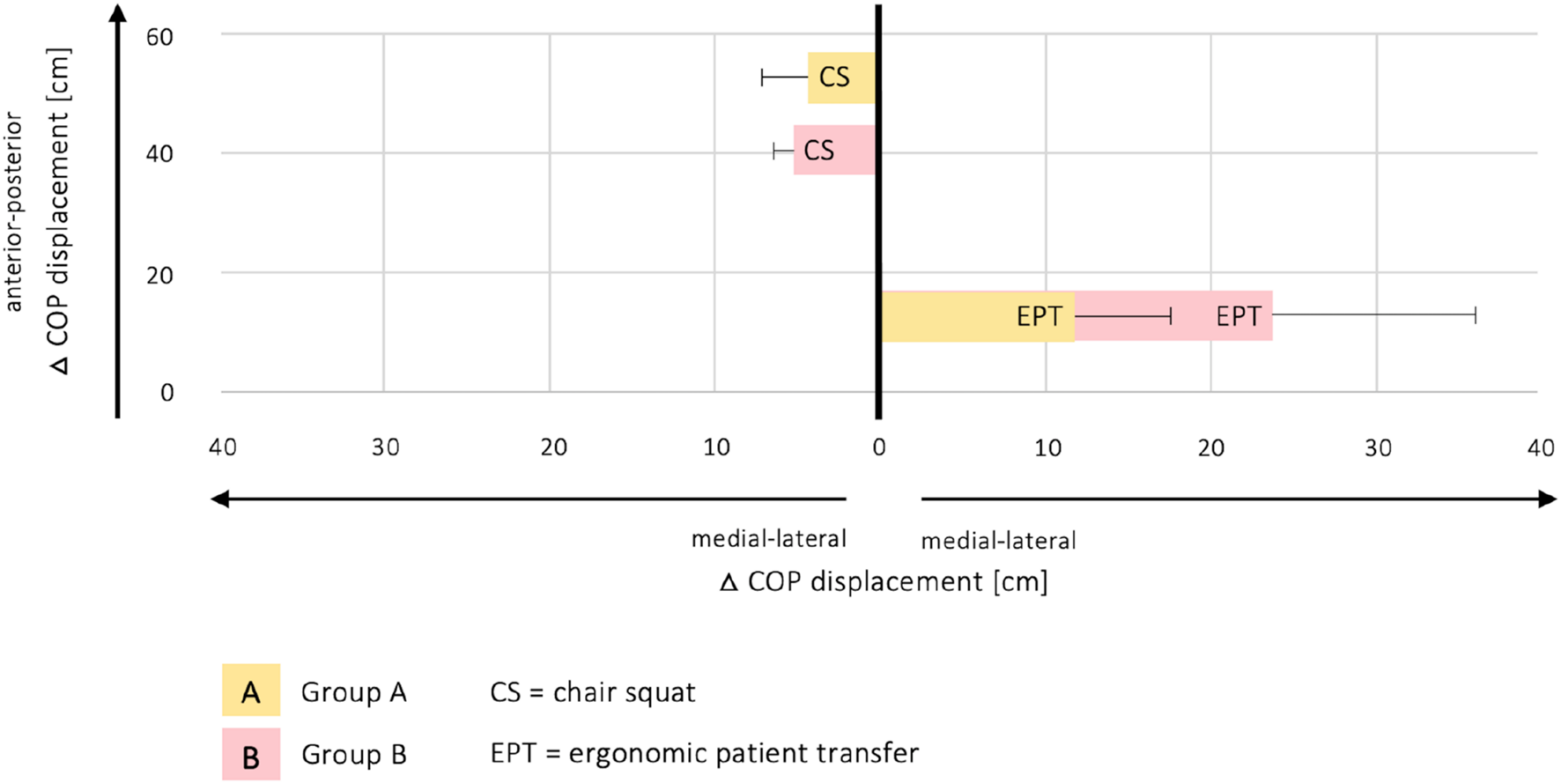
Representation of the medial–lateral COP displacements for both groups and tasks as a function of anterior–posterior COP displacement. The results are presented as means ± standard deviations. Comparisons of each group’s two-sided Wilcoxon signed-rank test reach statistical significance in the anterior–posterior direction of the CS and EPT (Supplementary Material, Table 4). The inter-group comparison showed no statistical significance (Supplementary Material, Table 3).

We also analyzed medial–lateral COP displacements in the CS and EPT as a function of anterior–posterior COP displacement (Fig. 4). The left abscissa shows the medial–lateral COP displacement for the CS, and the right abscissa represents that for EPT. The ordinate depicts anterior–posterior COP displacements. The inter-group comparison was not statistically significant (Supplementary Material, Table 3). Intra-group comparisons in the anterior–posterior direction (Supplementary Material, Table 4) represented the main differences. Differences in the medial–lateral direction were present as well (Fig. 4), but were not statistically significant (Supplementary Material, Tables 3 and 4).

#### Surface electromyography

**Figure 5:**
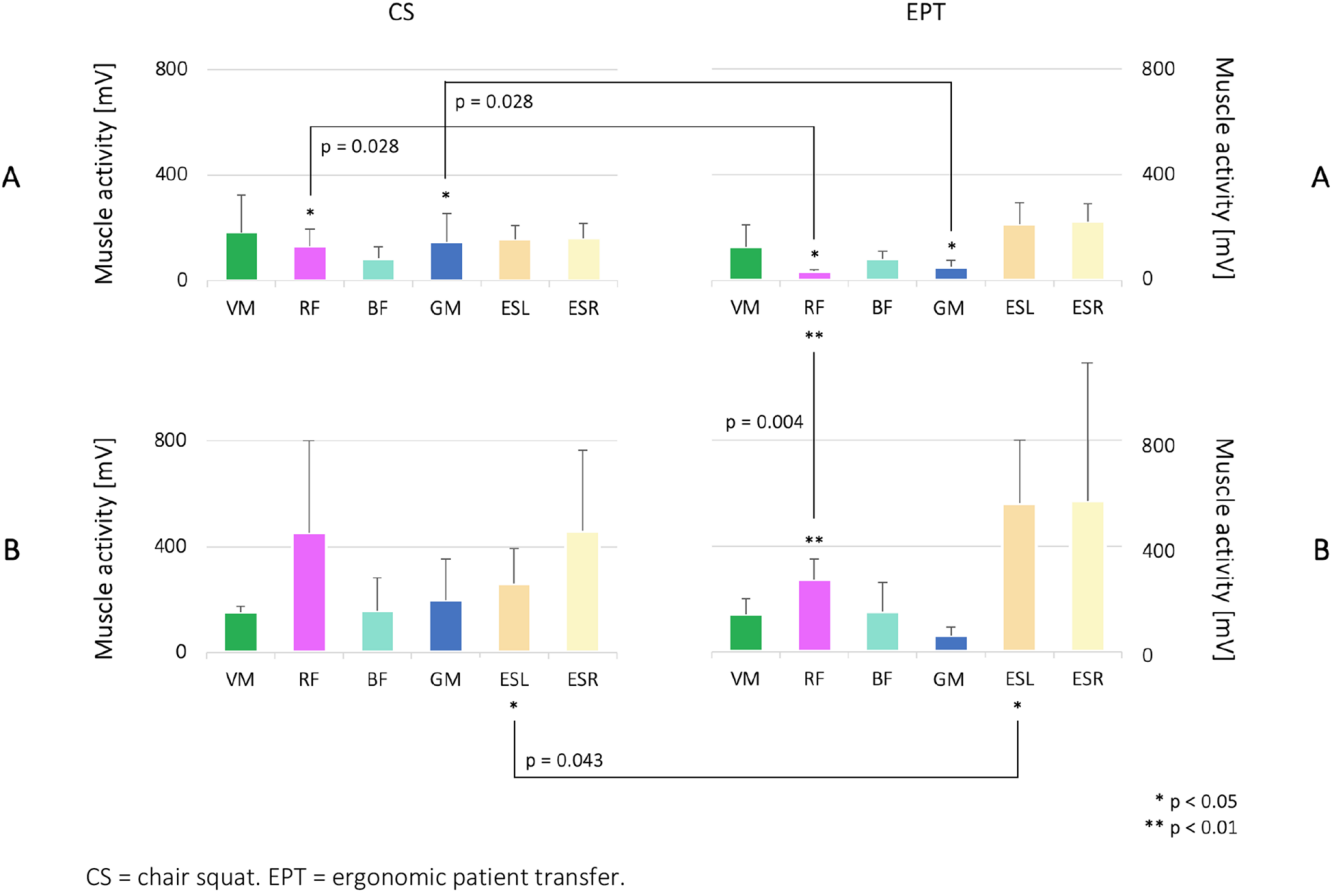
RMS muscle activity data of vastus medialis (VM), rectus femoris (RF), biceps femoris (BF), gluteus maximus (GM), left erector spinae (ESL), and right erector spinae (ESR). The results are presented as means ± standard deviations, including significant comparisons of each group’s two-sided Wilcoxon signed-rank test (Supplementary Material, Table 5). Significant results of the Mann–Whitney test for inter-group comparison are presented in Supplementary Material, Table 6.

Different muscle activation patterns were present for both groups and tasks (Fig. 5). When squatting down in CS and EPT, group **A** demonstrated similar activation patterns with the highest value in VM, followed by RF and BF. There were significant differences between both performances for RF (*p* = 0.028) and GM (*p* = 0.028) (Fig. 5 and Supplementary Material, Table 5). In comparison to group **A**, group **B** demonstrated predominantly higher activation of the back extensors (ESL/ESR) and RF during CS and EPT. Significant differences between both tests were also present for ESL (*p* = 0.043) (Fig. 5 and Supplementary Material, Table 6). An inter-group comparison of both tasks indicated that RF and ESR muscle activity were predominantly higher in the CS of group **B**, but without reaching statistical significance (Fig. 5 and Supplementary Material, Table 6). Given the results for EPT, RF muscle activity increased significantly (*p* = 0.004) in group **B** (Fig. 5 and Supplementary Material, Table 6).

To retrospectively analyze the frequency distribution of the variables according to RF activation in CS and EPT and to provide an assessment of the predictive capability, we used a 2 × 2 contingency table for statistical verification (Supplementary Material, Fig. 1). For this purpose, we set RF < VM (group **A**) or RF > VM (group **B**) as a condition. Each condition was verified for each participant. We calculated row and column-wise parameters based on the table, including positive and negative predictive values, sensitivity, and specificity. The accuracy rate is calculated based on all four parameters. The predictive capability of the CS was measured with an accuracy rate of 73% (Supplementary Material, Fig. 2).

## Discussion

This cross-sectional pilot study investigated kinetic and EMG assessments in our laboratory^25^ to assess the CS and EPT ability of nurses. We hypothesized that the CS is a potential predictor of nurses’ physical capabilities in occupational duties such as EPT. The study confirmed our assumptions that the analysis of kinetic muscle activation behavior in the lower limbs and spine indeed also sheds light on patterns of health-enhancing and compensational strategies, as we discuss below.

Squatting down from a stable standing position is essential for daily occupational activities in the nursing field. These posture changes require extensive joint moments in the lower limbs and trunk, including precise, dynamic balance control, which affects mass load on various parts of the body. While displacing the center of mass downward, adequate body mechanics are essential for countering bodyweight eccentrically; this is supported by lower limb and spine muscle strength. In a comparison focusing on eccentric knee flexion (and thus concentric resistance of knee extension) when squatting down in CS and EPT, similar activation patterns with the highest value in VM, followed by RF and BF, were found for group **A** (Fig. 5). This type of muscle activity pattern is identical to previous measurements^31^ as well as to literature findings^32–35^. Compared to the CS performance, an evident increase in ESL/ESR mean muscle activity is shown in the EPT task for both groups **A** and **B**. This may be due to increased trunk flexion during this task (Fig. 2) because of increased medial–lateral COP displacement (Fig. 4) and postural sway (Fig. 3). Literature findings have indicated that the spine is supported by its ligaments in a fully extended position, reducing spine muscle activity^36^. When looking at the kinetic data and the muscle activation behavior of group **B**, compensational strategies in both CS and EPT drove the highest degree of asymmetry. This was indicated by increased mean ESL/ESR activity for balance control, a significant increase in the torsion, and an increase in lateral flexion of the trunk when moving the patient simulator (Figs. 2 and 5). Moreover, subjects’ COP displacement and postural sway increased in the medial–lateral direction in the EPT compared to the CS (Figs. 3 and 4). This also affected RF and BF muscle activation behavior, as these knee extensors and flexors are essential to enabling balance, counteracting the body’s postural sway by muscular contraction^37^. Compared to group **A**, group **B** showed greater mean muscle activity in the BF in both tasks, though this failed to reach statistical significance. In contrast, there was a highly significant difference in RF muscle activation between group **A** and group **B** (Fig. 5, EPT). Given that hip flexion redistributes moments from the hip to the knee or ankle joints^38^, appropriate motion strategies increase the knee’s extensor moment and thus muscle activity. This is evident when discussing the activation of the RF and BF muscles. While RF was activated eccentrically, BF activation was concentric when counteracting bodyweight and gravity. Results from previous literature indicate that the relative amount of muscular strength to maintain balance increases when a larger mass is controlled^39^. In addition, greater postural sway requires greater muscular strength to avoid the subject losing balance^39^. Our results corroborate this. Looking at the physical characteristics of each group, that may have influenced our results, the need for individual intervention programs is highlighted. Group **A** participants had a mean age of 28.17 ± 3.43 years, a mean bodyweight of 68.17 ± 11.62 kg, and a mean body height of 1.72 ± 0.09 m. Group **B** participants had a mean age of 39.80 ± 13.39, a mean bodyweight of 78.71 ± 24.10 kg, and a mean body height of 1.72 ± 0.09 m. Compared to group **A**, group **B** had an increased postural sway in the medial–lateral direction, as well as increased activity in the knee extensor (RF), knee flexor (BF), and lumbar erector (ESL/ESR) muscles due to compensational strategies of balance control during both CS and EPT. These findings imply that adapted trunk flexion due to asymmetric behavior that disrupts balance and control of the movement affects biomechanics and muscle activity.

In addition, generally poor fitness and obesity are other examples of occupational risk factors. Obesity can lead to an anteriorly tilted pelvis and lumbar lordosis; this affects the musculature, including its force couple relationship in the sacroiliac joints^40^. Here, the pelvis’ anterior rotation is caused by concentric muscular contraction of the RF. This eccentrically lengthens BF and GM, thus impairing the force couple relationship, which subsequently causes muscle strains and MSD. To counteract this, concentric strengthening of BF and GM is essential to reverse the anterior tilt and neutrally realign the pelvis. Muscles that need to be stretched include the knee extensor/hip flexor muscles and lower back muscles. Muscles that need to be strengthened include the hip extensors. For this purpose, closed kinetic chain exercises, e.g., squatting performances, have been recommended^40^. Two insights would help minimize the risk of developing MSD in the nursing profession by ensuring good health and fitness: First, workplace-specific training exercises individually adapted to the high physical demand in care would significantly aid nurses; and second, this training could be used as a diagnostic tool to identify existing deficits.

To retrospectively analyze the frequency distribution of the variables according to RF activation in CS and EPT and to assess the predictive capability, we used a 2 × 2 contingency table for statistical verification (Supplementary Material, Fig. 1). For this purpose, we set RF < VM (group **A**) and RF > VM (group **B**) as target conditions. Each condition was verified for each participant. The sensitivity and specificity of the CS as a predictor for nurses’ capability in EPT based on group classification were measured with a rate of 80% and 67%, respectively (Supplementary Material, Fig. 2). These values indicate the concordance of the test concerning the chosen conditions. The rate of positive predicted values was 67%, and the rate of negative predicted values was 80% (Supplementary Material, Fig. 2). That means 4 out of 6 participants were predicted correctly for the condition RF < VM (group **A**), and 4 out of 5 participants were predicted correctly for the condition RF > VM (group **B**). The ability of the CS as a predictor for nurses’ capability in EPT based on group classification was measured with an accuracy rate of 73% (Supplementary Material, Fig. 2). In other words, 8 out of 11 participants were predicted correctly based on the target conditions. The results indicate that the CS could be a potential predictor of nurses’ physical capabilities with respect to EPT. Especially group **B** showed potential deficits in CS concerning muscle activity that also carried over to EPT, e.g., higher postural sway, including higher anterior moments, was accompanied by strong contraction of RF and ESL/ESR, resulting in muscle activation in BF to counteract these stresses.

This cross-sectional pilot study served as data collection to calculate the number of cases for future studies using effect size calculation. The reported findings represent initial results. Future studies with larger sample sizes should investigate whether consistently conducted CS performance as a training intervention affects the balance-control ability of nurses in CS and EPT tasks. Basically, the CS is a potential training intervention as it can be performed at a sufficient intensity for nurses’ individual concentric and eccentric bodyweight training and integrated into daily routines.

By quantifying nurses’ spine and lower-limb muscle activity during the CS and EPT, we contribute to the ongoing research of physical relief in care. Although physical performance declines with age and thus the incidence of MSD increases, it is essential to note that younger subjects also struggle with these problems^41^. Lower back pain occurs most frequently in the age group between 30 and 50 years of age and is equally observed in men and women^9,14^. It is also clear from prognoses that the number of people with lower back pain will increase^4^.

## Limitations and future research directions

The muscle activation data indicates a relatively large standard deviation, especially in group** B**. Although participants’ skin was adequately prepared, and electrodes were placed according to SENIAM guidelines^42^, crosstalk from other muscle groups may have affected EMG signaling. For example, muscle activation of the vastus intermedius may have contaminated the RF signal. Also, we did not test for muscle fatigue. Previous literature^27^ analyzed muscular activity and fatigue of healthy adults during CS tests under various speeds and repetitions using surface EMG in the lower limb and trunk muscles. Significant differences in fatigue of VM between these different test scenarios were recorded ^27^. It turned out that the VM plays the main role in the execution of squats, which corresponds to our findings when looking at lower limb muscle activity patterns of group **A**. In future research, muscular fatigue could be another relevant topic—especially given manual patient handling techniques that impact the musculoskeletal system. Moreover, we did not control for joint kinetics and its relation to the observed muscle activation. Nor did we control for action forces transmitted from participants to the bed in the EPT tasks. These topics could be relevant in future research.

One crucial issue is that this study was conducted with a small sample size due to the COVID-19 pandemic. The results and statistical analyses should therefore be interpreted with caution due to the possibility of a Type II error. The reported findings represent initial results that require larger sample sizes for future confirmation.

## Conclusion

The findings of our cross-sectional pilot study imply that adapted trunk flexion during CS and EPT activities due to asymmetric behavior (that disrupts balance and control of the conducted movement) affects the biomechanics and muscle activity of lower limb and spine muscles. It was therefore possible to distinguish two kinds of movement patterns. The relative amount of muscular activity to maintain equilibrium increases when a larger mass needed to be controlled. In addition, a larger postural sway requires greater knee extensor, knee flexor, and lumbar erector activity for balance control. These findings provide initial insights into biomechanics and muscle activity in the CS and EPT duties of nurses, thus identifying patterns of health-enhancing and compensational strategies. For nurses' support through worksite-tailored exercise programs like implementing squat training to strengthen the lower limb and spine muscles, using the CS is a promising predictor of nurses’ physical ability in occupational tasks like EPT, e.g., increased activity of RF is an indicator of increased hip flexion; therefore, for high torsional forces affecting the sacroiliac joints. In sum, existing difficulties during CS performance can be specifically targeted to prevent potential health risks during EPT. We contribute to the ongoing research on the physical relief of nurses through functional testing, providing interdisciplinary research knowledge for conceiving worksite-tailored exercises. Our aim is to individually adapt those training sessions to the high physical stresses and strains in the nursing profession.

## Supplementary Information

Supplementary Information.

## Data Availability

The data that support the findings of this study are available from the corresponding author upon reasonable request.
