# Supplementary material for: Chair squat performance as a potential predictor of nurses’ physical capabilities in ergonomic patient transfers
Source: Sci Rep. 2023 Feb 17;13:2825. doi: 10.1038/s41598-023-29968-0 (PMC9936124; doi:10.1038/s41598-023-29968-0)
Supplement: Supplementary file 1 — Supplementary Information. [file 41598_2023_29968_MOESM1_ESM.pdf]

Chair squat performance as a potential predictor of nurses' physical capabilities in ergonomic patient transfers

Supplementary Material

Anna Brinkmann[1,*], Christian Kowalski[1], Sandra Lau[2], Ole Meyer[1], Rebecca Diekmann[1] and Andreas Hein[1]

[1] Assistive Systems and Medical Device Technology, Carl von Ossietzky University of Oldenburg, 26129 Oldenburg, Germany

[2] Geriatric Medicine, Carl von Ossietzky University of Oldenburg, 26129 Oldenburg, Germany

[*] Corresponding author: anna.brinkmann1@uni-oldenburg.de

Chair squat vs. ergonomic patient transfer

Force plate - moments (M)

Table 1. Inter-group comparison of medial-lateral and torsional moments for both groups (A, B) and tasks. P-values and effect sizes (*r*) are presented for non-parametric statistical testing using the Mann-Whitney test.

	Ø Moments [Nm]			
	A	B	*p-value*	*r*
CS				
$M_{medial\text{-}lateral}$	287.334 ± 209.617	348.468 ± 151.610	0.715	0.110
$M_{torsional}$	56.987 ± 62.733	82.077 ± 73.608	0.584	0.165
$M_{anterior\text{-}posterior}$	1637.378 ± 411.288	2016. 512 ± 881.329	0.715	0.110
EPT				
$M_{medial\text{-}lateral}$	443.951 ± 384.753	967.688 ± 828.836	0.201	0.385
$M_{torsional}$	137.983 ± 101.086	247.249 ± 123.577	0.465	0.220
$M_{anterior\text{-}posterior}$	2059.848 ± 341.510	2257.948 ± 1169.153	0.144	0.441

M = moments. CS = chair squat. EPT = ergonomic patient transfer.

Table 2. Intra-group comparison of medial-lateral and torsional moments for both groups (A, B) and tasks. P-values and effect sizes (*r*) are presented for non-parametric statistical testing using the two-sided Wilcoxon signed-rank test.

	Ø Moments [Nm]			
	CS	EPT	*p-value*	*r*
A				
$M_{medial\text{-}lateral}$	287.334 ± 209.617	443.951 ± 384.753	0.463	0.221
$M_{torsional}$	56.987 ± 62.733	137.983 ± 101.086	0.116	0.474
$M_{anterior\text{-}posterior}$	1637.378 ± 411.288	2059.848 ± 341.510	* 0.028	0.664
B				
$M_{medial\text{-}lateral}$	348.468 ± 151.610	967.688 ± 828.836	0.138	0.447
$M_{torsional}$	82.077 ± 73.608	247.249 ± 123.577	* 0.043	0.610
$M_{anterior\text{-}posterior}$	2016. 512 ± 881.329	2257.948 ± 1169.153	0.686	0.122

M = moments. CS = chair squat. EPT = ergonomic patient transfer.

Force plate - center of pressure (COP)

Table 3. Inter-group comparison of the mean COP displacement in medial-lateral and anterior-posterior directions for both groups (A, B) and tasks. P-values and effect sizes (*r*) are presented for non-parametric statistical testing using the Mann-Whitney test.

	Δ COP displacement [cm]			
	A	B	*p-value*	*r*
CS				
$COP_{medial-lateral}$	3.753 ± 2.638	4.580 ± 1.095	1.000	0.000
$COP_{anterior-posterior}$	52.425 ± 15.351	40.546 ± 15.314	0.537	0.220
EPT				
$COP_{medial-lateral}$	11.185 ± 6.370	23.082 ± 12.804	0.931	0.055
$COP_{anterior-posterior}$	12.432 ± 4.785	12.604 ± 7.363	0.126	0.495

COP = center of pressure. CS = chair squat. EPT = ergonomic patient transfer.

Table 4. Intra-group comparison of the mean COP displacement in medial-lateral and anterior-posterior directions for both groups (A, B) and tasks. P-values and effect sizes (*r*) are presented for non-parametric statistical testing using the two-sided Wilcoxon signed-rank test.

	Δ COP displacement [cm]			
	CS	EPT	*p-value*	*r*
A				
$COP_{medial-lateral}$	3.753 ± 26.378	11.185 ± 6.370	0.116	0.642
$COP_{anterior-posterior}$	52.425 ± 15.351	12.432 ± 4.785	* 0.028	0.899
B				
$COP_{medial-lateral}$	4.580 ± 1.095	23.082 ± 12.804	0.080	0.784
$COP_{anterior-posterior}$	40.546 ± 15.314	12.604 ± 7.363	* 0.043	0.905

COP = center of pressure. CS = chair squat. EPT = ergonomic patient transfer.

Surface electromyography

Table 5. Intra-group comparison of mean RMS muscle activity data for each muscle: vastus medialis (VM), rectus femoris (RF), biceps femoris (BF), gluteus maximus (GM), left erector spinae (ESL), and right erector spinae (ESR). P-values and effect sizes (*r*) are presented for non-parametric statistical testing using the two-sided Wilcoxon signed-rank test.

Ø Muscle activity [mV]				
	CS	EPT	*p-value*	*r*
A				
VM	183.637 ± 141.011	127.272 ± 84.176	0.600	0.214
RF	130.472 ± 63.938	35.008 ± 5.753	* 0.028	0.899
BF	83.482 ± 44.571	81.049 ± 29.367	0.753	0.128
GM	147.522 ± 107.292	51.958 ± 24.146	* 0.028	0.899
ESL	156.872 ± 52.069	213.883 ± 79.668	0.173	0.556
ESR	161.714 ± 54.946	223.097 ± 66.127	0.686	0.181
B				
VM	151.974 ± 22.776	141.132 ± 59.558	0.893	0.060
RF	453.796 ± 351.071	273.567 ± 79.505	0.345	0.422
BF	158.207 ± 125.343	151.890 ± 111.671	0.715	0.183
GM	198.231 ± 155.974	61.634 ± 31.305	0.138	0.663
ESL	260.971 ± 132.249	562.748 ± 243.006	* 0.043	0.905
ESR	459.602 ± 306.301	572.658 ± 523.187	0.080	0.784

CS = chair squat. EPT = ergonomic patient transfer.

Table 6. Inter-group comparison of mean RMS muscle activity data for each muscle: vastus medialis (VM), rectus femoris (RF), biceps femoris (BF), gluteus maximus (GM), left erector spinae (ESL), and right erector spinae (ESR). P-values and effect sizes (*r*) are presented for non-parametric statistical testing using the Mann-Whitney test.

Ø Muscle activity [mV]				
	A	B	*p-value*	*r*
CS				
VM	183.637 ± 141.011	151.974 ± 22.776	0.792	0.110
RF	130.472 ± 63.938	453.796 ± 351.071	0.082	0.551
BF	83.482 ± 44.571	158.207 ± 125.343	0.352	0.337
GM	147.522 ± 107.292	198.231 ± 155.974	0.662	0.165
ESL	156.872 ± 52.069	260.971 ± 132.249	0.082	0.551
ESR	161.714 ± 54.946	459.602 ± 306.301	0.310	0.363
EPT				
VM	127.272 ± 84.176	141.132 ± 59.558	0.429	0.281
RF	35.008 ± 5.753	273.567 ± 79.505	** 0.004	0.826
BF	81.049 ± 29.367	151.890 ± 111.671	0.082	0.551
GM	51.958 ± 24.146	61.634 ± 31.305	0.792	0.110
ESL	213.883 ± 79.668	562.748 ± 243.006	0.052	0.605
ESR	223.097 ± 66.127	572.658 ± 523.187	0.056	0.627

CS = chair squat. EPT = ergonomic patient transfer.

Assessment for predictive capabilities

2x2 contingency table

		OUTCOME		
		**Positive**	**Negative**	
PREDICTION	**Condition 1**	a<br>True positive	b<br>False positive	**Positive predicted values**<br>[a/(a+b)]
	**Condition 2**	c<br>False negative	d<br>True negative	**Negative predicted values**<br>[d/(c+d)]
		**Sensitivity**<br>[a/(a+c)]	**Specificity**<br>[d/(b+d)]	**Accuracy**<br>[(a+d)/(a+b+c+d)]

Figure 1. 2x2 contingency table for displaying and evaluating the outcome of predictions. True positive and true negative values are depicted in green and represent the correct classifications. The values outside the green diagonal represent the errors. The equations for the most used metrics are also given. Sensitivity and specificity indicate the concordance of a test concerning a chosen condition [1]. The positive and negative predicted values indicate the probability that a test can successfully identify whether participants do or do not fulfill a target condition based on their test results [1]. Accuracy indicates the number of correct classifications, thus the number of true positive and true negative values.

				EPT		
			#	**RF < VM**	**RF > VM**	
CS	A	**RF < VM**	6	4	2	**Positive predicted values**<br>**67%**<br>4 out of 6 were predicted correctly
	B	**RF > VM**	5	1	4	**Negative predicted values**<br>**80%**<br>4 out of 5 were predicted correctly
		Σ	11	**Sensitivity**<br>**80%**<br>4 out of 5 were predicted correctly	**Specificity**<br>**67%**<br>4 out of 6 were predicted correctly	**Accuracy**<br>**73%**<br>8 out of 11 were predicted correctly

CS = chair squat. EPT = ergonomic patient transfer. RF = rectus femoris. VM = vastus medialis.

Figure 2. 2x2 contingency table for displaying and evaluating the outcome of predictions of nurses' physical capabilities in EPT based on the CS performance. The conditions are RF < VM (group A) and RF > VM (group B). Each condition was verified for each participant. Metrics were calculated row and column-wise based on the table and are presented as percentages.

References

[1] Trevethan, R. Sensitivity, specificity, and predictive values: foundations, pliabilities, and pitfalls in research and practice. Front. Public Health. 5, 2017. doi: 10.3389/fpubh.2017.00307
